# Antiflammin-1 attenuates bleomycin-induced pulmonary fibrosis in mice

**DOI:** 10.1186/1465-9921-14-101

**Published:** 2013-10-08

**Authors:** Wei Liu, Jing Wan, Jian-Zhong Han, Chen Li, Dan-Dan Feng, Shao-Jie Yue, Yan-Hong Huang, Yi Chen, Qing-Mei Cheng, Yang Li, Zi-Qiang Luo

**Affiliations:** 1 Department of Physiology, Xiangya School of Medicine, Central South University, 110 Xiangya Road, Changsha 410078, PR China; 2 Department of Pediatrics, Xiangya Hospital of Central South University, Changsha 410008, PR China; 3 Department of Physiology, ChangZhi Medical College, Changzhi 046000, PR China

**Keywords:** Bleomycin, Pulmonary fibrosis, Antiflammin-1, Uteroglobin receptor

## Abstract

**Background:**

Antiflammin-1 (AF-1), a derivative of uteroglobin (UG), is a synthetic nonapeptide with diverse biological functions. In the present study, we investigated whether AF-1 has a protective effect against bleomycin-induced pulmonary fibrosis.

**Methods:**

C57BL/6 mice were injected with bleomycin intratracheally to create an animal model of bleomycin-induced pulmonary fibrosis. On Day 7 and Day 28, we examined the anti-inflammatory effect and antifibrotic effect, respectively, of AF-1 on the bleomycin-treated mice. The effects of AF-1 on the transforming growth factor-beta 1 (TGF-β1)-induced proliferation of murine lung fibroblasts (NIH3T3) were examined by a bromodeoxycytidine (BrdU) incorporation assay and cell cycle analysis.

**Results:**

Severe lung inflammation and fibrosis were observed in the bleomycin-treated mice on Day 7 and Day 28, respectively. Administration of AF-1 significantly reduced the number of neutrophils in the bronchoalveolar lavage fluid (BALF) and the levels of tumor necrosis factor-alpha (TNF-α) and interleukin-1 beta (IL-1β) in the lung homogenates on Day 7. Histological examination revealed that AF-1 markedly reduced the number of infiltrating cells on Day 7 and attenuated the collagen deposition and destruction of lung architecture on Day 28. The hydroxyproline (HYP) content was significantly decreased in the AF-1-treated mice. *In vitro*, AF-1 inhibited the TGF-β1-induced proliferation of NIH3T3 cells, which was mediated by the UG receptor.

**Conclusions:**

AF-1 has anti-inflammatory and antifibrotic actions in bleomycin-induced lung injury. We propose that the antifibrotic effect of AF-1 might be related to its suppression of fibroblast growth in bleomycin-treated lungs and that AF-1 has potential as a new therapeutic tool for pulmonary fibrosis.

## Background

Pulmonary fibrosis is a progressive disorder characterized by the excessive proliferation of fibroblasts and deposition of extracellular matrix, which destroy normal tissue architecture and function. The mechanisms of pulmonary fibrosis are not completely understood, and the effects of drugs on idiopathic pulmonary fibrosis (IPF), a fatal respiratory disease in humans, are not satisfactory [1]. Therefore, it is crucial to find new therapeutic strategies for pulmonary fibrosis.

Clara cells are secretory cells that are mainly distributed in terminal respiratory bronchioles and the respiratory bronchiole epithelium. Clara cell secretory protein (CCSP) is mainly secreted from Clara cells. CCSP is often called uteroglobin (UG) because UG/CCSP was initially found in the endometrium of early-pregnancy-phase rabbits. UG/CCSP is a small secretory protein that has a variety of pharmacological and physiological effects *in vitro* and *in vivo*. UG/CCSP has potent anti-inflammatory, antichemotactic and immunomodulatory properties [2,3] and also inhibits the proliferation of various cell lines [4-6]. In our previous study, we found that after bleomycin treatment, Clara cell-ablated mice that were exposed to naphthalene vapor exhibited more severe lung pathology and collagen deposition [7]. Lee and colleagues also reported that UG knockout mice were extraordinarily sensitive to bleomycin and were highly susceptible to developing pulmonary fibrosis [8]. These results suggest that UG, as an endogenous protective factor, plays an important role in inhibiting pulmonary fibrosis.

Antiflammin-1 (AF-1), a peptide with the sequence methionine - glutamine - methionine - lysine - lysine - valine - leucine - aspartic acid - serine (MQMKKVLDS), is equivalent to the carboxyl-terminal part of the third α-helix of uteroglobin and has biological characteristics similar to those of its parent protein [9,10]. AF-1 has a wide range of functions. For example, *in vitro*, AF-1 can inhibit leukocyte adhesion and function, suppress macrophage activation, reduce platelet aggregation, prevent mast cell degranulation, block lymphatic vessel contraction and decrease inflammatory mediator production and release [11-15]. *In vivo*, AF-1 has highly potent anti-inflammatory activity in different animal models, tissues and organs [9,16-18]. Despite progress in recent decades, our understanding of the biological properties of AF-1 remains incomplete. Although AF-1 has been explored by numerous groups, there is no research on this molecule in the field of fibrosis.

Because UG plays an important role in inhibiting pulmonary fibrosis, we sought to determine whether AF-1 could also protect against pulmonary fibrosis. We found that AF-1 attenuated bleomycin-induced pulmonary fibrosis. Our findings suggest that AF-1 has potential as a new therapeutic tool for pulmonary fibrosis.

## Materials and methods

### Ethics statement

The Ethics Committee Institute of Clinical Pharmacology of the Central South University (Changsha, China) approved the experiments, which were performed in accordance with National Institutes of Health guidelines. All surgery was performed using anesthesia, and all efforts were made to minimize suffering.

### Animal treatment protocol

Adult male C57BL/6 mice were obtained from the laboratory animal unit of Central South University. After being anesthetized, mice were intratracheally injected with 50 μL of bleomycin (5 mg · kg^-1^) on Day 0. For time course experiments, lung samples were collected on Days 0, 3, 7, 10, 14, 21 and 28 for further analysis.

AF-1 was synthesized according to the standard solid-phase method by the Shanghai Shenggong Bioengineering Company (China) and stored at -20°C until use [19]. To investigate the antifibrotic effect of AF-1, mice were randomly divided into four groups: (1) intratracheal saline plus intraperitoneal saline (control group); (2) intratracheal saline plus intraperitoneal AF-1 (AF-1 group); (3) intratracheal bleomycin (BLM) plus intraperitoneal saline (BLM group); (4) intratracheal BLM plus intraperitoneal AF-1 (BLM + AF-1 group). For the preventive antifibrotic treatment, AF-1 (2 mg · kg^-1^ · day^-1^) was administered from Day 0 to Day 27, and lung samples were collected on Day 28. For the therapeutic antifibrotic treatment, AF-1 (2, 5 or 10 mg · kg^-1^ · day^-1^) was administered from Day 11 to Day 27, and mice were sacrificed on Day 28. To determine whether continuous treatment with AF-1 (2 mg · kg^-1^ · day^-1^) could inhibit the inflammatory response in the bleomycin mouse model, AF-1 was injected from Day 0 to Day 6, and mice were sacrificed on Day 7.

### ELISA for cytokine measurements

An enzyme-linked immunosorbent assay (ELISA) was used to determine the concentrations of several cytokines (tumor necrosis factor-alpha (TNF-α), interleukin-1 beta (IL-1β) and IL-6) in the lungs. After a thoracotomy, the lungs were removed and homogenized in phosphate-buffered saline (PBS, pH 7.4) containing protease inhibitors (Roche, Germany). The lung homogenates were centrifuged at 10,000 × *g* to remove insoluble debris. The supernatants were assayed with ELISA kits according to the manufacturer’s instructions (Invitrogen, CA).

### Bronchoalveolar lavage

The mice were anesthetized, and after exposure of the trachea, a plastic cannula was inserted into the trachea. A syringe was used to inject 1 mL of 0.9% saline solution into the lungs and was then withdrawn. This injection procedure was repeated five times. After the number of cells in the bronchoalveolar lavage fluid (BALF) was counted, BALF samples were centrifuged, and the cell pellets were resuspended in PBS. Then, the cells were cytospun onto glass slides and stained with Wright’s-Giemsa Stain for cell classification.

### Hydroxyproline assay

The collagen content in the lung homogenates was examined by a hydroxyproline (HYP) assay (HYP kit from Nanjing Jiancheng Bioengineering Company, China). All steps of the HYP assay were performed according to the manufacturer’s instructions. The absorbance of each sample at 550 nm wavelength was read by a microplate reader (Thermo Fisher Scientific, USA).

### Histopathology

The lung samples were fixed in 4% buffered paraformaldehyde and embedded in paraffin. Sections were stained with hematoxylin-eosin and Masson’s trichrome. The Ashcroft score was used for the quantitative histologic analysis [20].

### Wet/Dry weight ratio assay

The wet/dry (W/D) method was used to measure pulmonary edema. After a thoracotomy, the lungs were collected and weighed before and after drying in the incubator at 60°C for 72 h.

### Cell line

Murine lung fibroblasts (NIH3T3) were obtained from the National Genetics Laboratory (Changsha, China). The cells were cultured in Dulbecco’s modified Eagle’s medium (DMEM) (Gibco) supplemented with 2 mM L-glutamine (Sigma, USA) and 10% fetal bovine serum (FBS) (Gibco).

### Antibody preparation

The peptides corresponding to mouse UG receptor (Accession No.AY052398.1, GI:23707077) were synthesized with free N and C terminus according to the standard solid phase method by Jingmei bioengineering company (Beijing, China) for experimental immunization [19]. Adult New Zealand white rabbits were injected subcutaneously with 2.5 mg peptides in Complete Freund’s Adjuvant (CFA) (Sigma, USA) six times, every 3 weeks. Animals were bled before immunization and 8–10 days after each injection, and the serological response of immunized rabbits was tested by ELISA. The absorbance (optical density) at 405 nm (OD 405 nm) was measured using a microplate reader. The animals were sacrificed 60 days later (when a strong serological response was detected), and the specific antisera were finally obtained. The antisera were further purified using CNBr-activated Sepharose 4B (Pharmacia, USA) according to protocol provided by the manufacturer. Purity and reactivity of the antisera were checked by SDS-PAGE and ELISA, respectively. The antisera were then stored at -70°C until use, as described for UG receptor antibody.

### Bromodeoxycytidine (BrdU) incorporation assay

NIH3T3 cells were seeded in 48-well plates and grown for 24 h. Before the experiments, the medium was changed to the incubation medium containing transforming growth factor-β1 (TGF-β1) (10 ng · mL^-1^) (RD Systems, USA) in the absence or presence of AF-1 (10 μM) and with or without anti-UG receptor antibody (50 μg · mL^-1^). Then, cells were cultured for an additional 12 h and were pulsed with BrdU for 2–6 h. Cell proliferation was assessed using a BrdU colorimetric cell proliferation ELISA kit (Roche, Germany). Similar experimental conditions were used in the following methods.

### Cell cycle analysis

The stages of the cell cycle were determined by flow cytometry. In brief, NIH3T3 cells were resuspended in a mixture of 0.3 mL of PBS and 0.7 mL of ice-cold ethanol. Propidium iodide (50 μg · mL^-1^) and ribonuclease A (20 μg · mL^-1^) were added to the mixture, which was then incubated overnight at 4°C. The cell cycle histograms were acquired on a FACSCalibur instrument using CELLQUEST software (Becton Dickinson) and were analyzed using ModFit LT V3.1 software (Verity Software House).

### Cyclin measurements

The protein levels of cyclin D1 and p27 were detected by flow cytometry. In brief, cells were resuspended in 0.5 mL of PBS, mixed with 0.5 mL of 4% paraformaldehyde and then permeabilized with Triton X-100 (Promega, USA). The permeabilized cells were stained with the phycoerythrin-conjugated antibody of anti-cyclin D1 or anti-p27 (Santa Cruz, USA). Single-color histograms were acquired on the FACSCalibur and analyzed using CELLQUEST software.

### Statistics

Comparisons were made using one-way analysis of variance followed by the Student-Newman-Keuls (SNK) test for multiple comparisons or using the nonparametric test (Mann–Whitney U test), depending on the distribution of the data. Survival rates were evaluated with a log-rank test over a period of 28 days. *P* ≤ 0.05 was considered significant.

## Results

### Time course of pulmonary fibrosis in bleomycin mouse model

**Figure 1:**
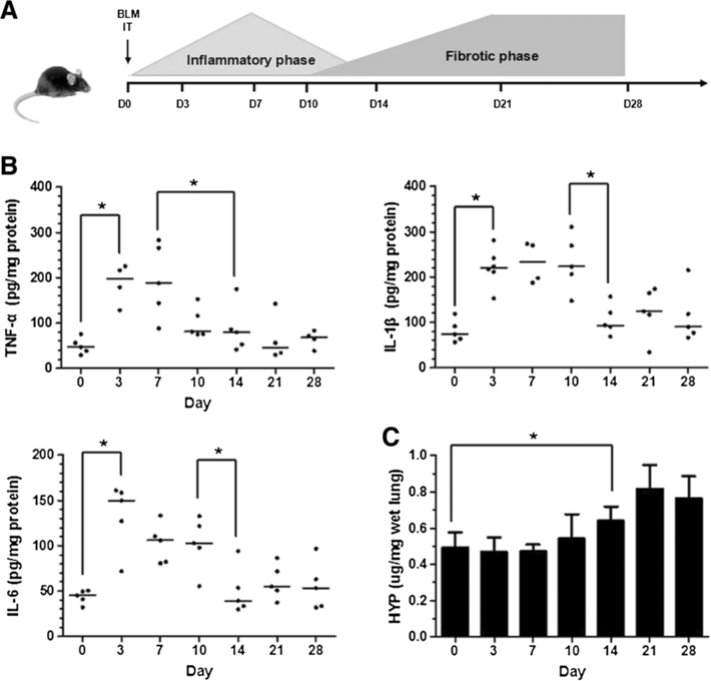
**Time Course for Bleomycin Induced Pulmonary Fibrosis. (A)**. Experimental design 1: Mice were injected with bleomycin (BLM) (5 mg · kg^-1^) intratracheally (IT) at D0. For time course experiment, at D0 (D0 = untreated control), D3, D7, D10, D14, D21 and D28, mice were killed and lung samples were collected for further analysis. **(B)**. The expression profile of inflammatory cytokines in the bleomycin mouse model. Bleomycin (5 mg · kg^-1^) was injected on D0. The lung tissues were collected at the indicated time points. The investigated inflammatory cytokines were tumor necrosis factor-alpha (TNF-α), interleukin-1beta (IL-1β) and IL-6. **(C)**. The expression profile of collagen was estimated by hydroxyproline (HYP) assay at the indicated time points. n = 4–7. Bars: median values (IL-1β, IL-6, TNF-α) or mean ± SD (HYP). *P ≤ 0.05.

C57BL/6 mice were intratracheally injected with bleomycin (5 mg · kg^-1^) on Day 0. The expression of inflammatory response markers in the lung tissue was measured by ELISA. The protein levels of TNF-α, IL-1β and IL-6 exhibited similar changes over the entire experimental period. The expression of pro-inflammatory cytokines increased rapidly during the first 3 days and remained high until Day 10. However, the expression of pro-inflammatory cytokines significantly decreased after Day 10 and continued to decrease until the end of the experiment (Figure 1B). The collagen content was tested using a HYP assay, which showed that there was no significant change in collagen deposition in the first 10 days. After Day 10, the collagen deposition increased rapidly and peaked on Day 21 (Figure 1C).

### The anti-inflammatory and antifibrotic activities of AF-1 in the bleomycin mouse model

**Figure 2:**
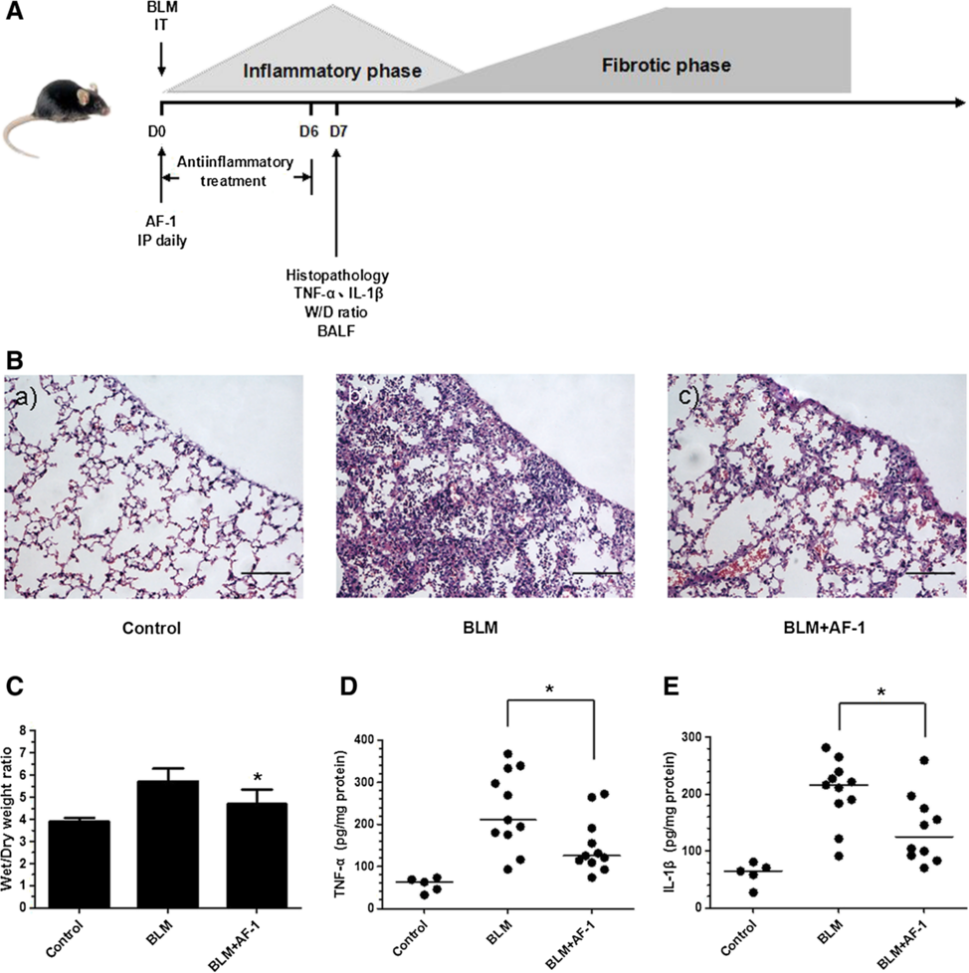
**The antiinflammatory effects of AF-1 on bleomycin-induced lung injury. (A)**. Experimental design 2: AF-1 (2 mg · kg^-1^ · day^-1^) was injected intraperitoneally from D0 to D6. On D7, mice were killed and lung samples were collected for further analysis. The degree of acute lung injury was assessed by H&E staining (×200, Scale bar = 100 μm) **(B)** and W/D ratio **(C)**. The levels of TNF-α **(D)** and IL-1β **(E)** in lung homogenate were quantified by ELISA. n = 5–11. Bars: median values (TNF-α and IL-1β) or mean ± SD (W/D). *P ≤ 0.05 vs BLM group.

Next, we sought to determine whether AF-1 also displays anti-inflammatory activity in response to bleomycin-induced lung injury. We administered AF-1 (2 mg · kg^-1^) to bleomycin-treated mice during the inflammatory phase of the model. A large increase in inflammatory cells was observed in the bleomycin-treated lungs on Day 7 (Figure 2B - b and Table 1). In contrast, following AF-1 treatment, the number of inflammatory cells was markedly reduced (Figure 2B - c and Table 1). Likewise, compared with the bleomycin-treated mice without AF-1, the mice treated with AF-1 showed a significant reduction in the lung W/D ratio (Figure 2C). We also found that AF-1 decreased the levels of TNF-α (Figure 2D) and IL-1β (Figure 2E) in bleomycin-treated mice. These data suggest that AF-1 inhibited the inflammatory response in this bleomycin-induced fibrosis model.

**Table 1:** Analysis of bronchoalveolar lavage

**Groups**	**Cells (×10**^**4**^**)**				**Cell classification (%)**		
	**Total**	**Macrophages**	**Neutrophils**	**Lymphocytes**	**Macrophages**	**Neutrophils**	**Lymphocytes**
Control	11.51 ± 3.98	11.0 ± 3.85	0.2 ± 0.1	0.25 ± 0.18	95.5 ± 1.97	1.8 ± 0.98	2.2 ± 1.53
BLM	55.93 ± 23.77	25.59 ± 15.28	18.08 ± 9.05	10.98 ± 5.18	44.95 ± 11.67	31.7 ± 8.13	20.5 ± 7.11
BLM + AF-1	32.81 ± 18.58*	22.55 ± 10.97	4.28 ± 4.54*	5.23 ± 3.36*	70.3 ± 8.28*	11.45 ± 5.2*	15.95 ± 4.33

AF-1 (2 mg · kg^-1^ · day^-1^) was injected intraperitoneally from D0 to D6. On D7, mice were killed and bronchoalveolar lavage was performed as described in METHODS. n = 5–10. Data are presented as mean **±** SD in each group. *P ≤ 0.05 vs BLM group.

**Figure 3:**
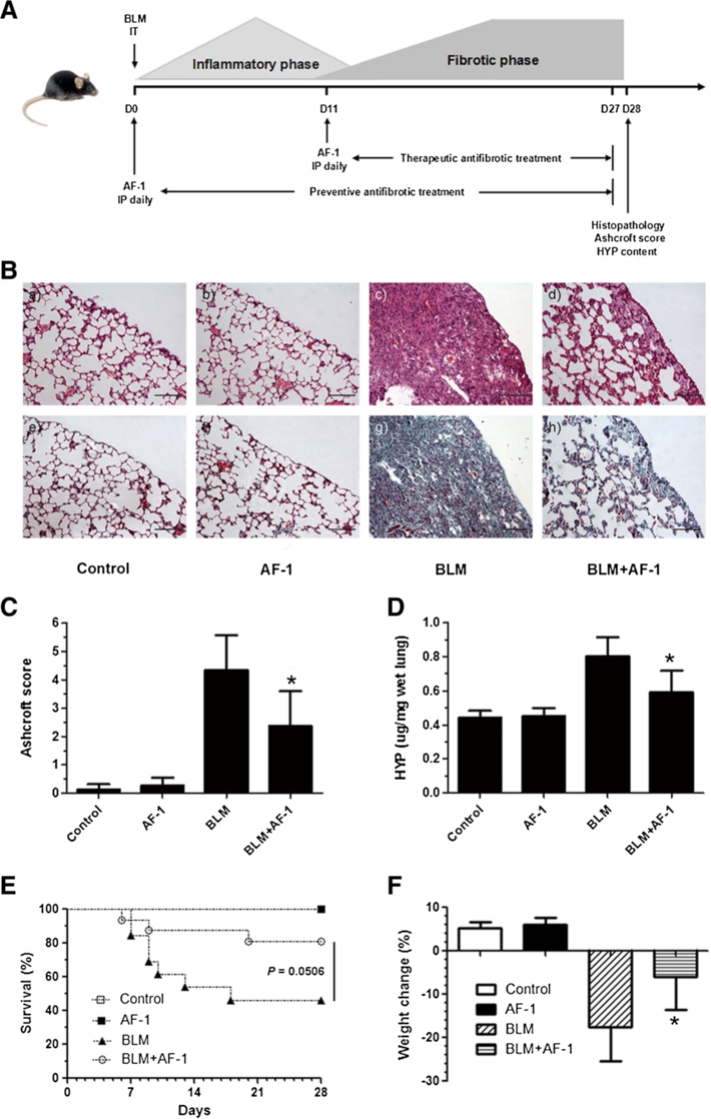
**The antifibrotic effects of preventive treatment with AF-1 on bleomycin-induced lung fibrosis. (A)**. Experimental design 3: The preventive treatment with AF-1 (2 mg · kg^-1^ · day^-1^) was performed from D0 to D27. The therapeutic treatment with AF-1 (2, 5 or 10 mg · kg^-1^ · day^-1^) was performed from D11 to D27. On D28, mice were killed and lung samples were collected for further analysis. **(B)** The histopathologic examination was determined by hematoxylin-eosin (H&E) staining (*upper panel* ) and Masson staining (*lower panel*) (×200, Scale bar = 100 μm). **(C)**. Ashcroft score was used to evaluate the degree of fibrosis. **(D)**. Collagen content was estimated by hydroxyproline assay. Survival rate **(E)** and Weight loss **(F)** were also monitored in the preventive treatment. n = 5–16. Bars: mean ± SD . *P ≤ 0.05 vs BLM group.

**Figure 4:**
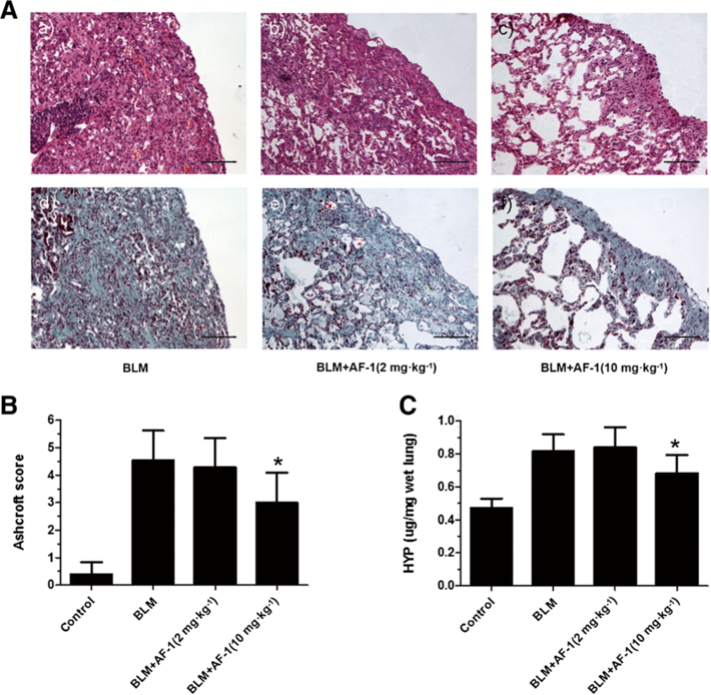
**The antifibrotic effects of therapeutic treatment with AF-1 on bleomycin-induced lung fibrosis. (A)** The histopathologic examination was determined by H&E staining (*upper panel* ) and Masson staining (*lower panel*) (×200, Scale bar = 100 μm). **(B)**. Ashcroft score was used to evaluate the degree of fibrosis. **(C)**. Collagen content was estimated by hydroxyproline assay. n = 5–7. Bars: mean ± SD . *P ≤ 0.05 vs BLM group.

Next, because the inflammatory response may play an important role in the development of bleomycin-induced pulmonary fibrosis, we used the report by Chaudhary et al. to design two treatment schedules. We used a preventative treatment and a therapeutic treatment to determine whether AF-1 reduces pulmonary fibrosis by preventing the progression of fibrosis or solely by interfering with the inflammatory response [21]. For the preventative treatment schedule, we treated bleomycin-induced mice with AF-1 (2 mg · kg^-1^) over the entire experimental period. Severe fibrosis was found using light microscopy in all bleomycin-treated mice (Figure 3B - c, g). In contrast, the lung fibrosis was markedly alleviated in the AF-1-treated mice (Figure 3B - d, h). Bleomycin induced an increase in the HYP content. Again, AF-1 (2 mg · kg^-1^) significantly reduced the HYP content (Figure 3D). We also examined the effect of AF-1 on the survival rate each day (Figure 3E) and the weight loss on Day 28 (Figure 3F) in bleomycin-treated mice. The preventative treatment with AF-1 (2 mg · kg^-1^) improved the survival rate (*P* = 0.0506) and the weight loss of mice injected with bleomycin. For the therapeutic treatment schedule, AF-1 (2, 5 or 10 mg · kg^-1^) was injected during the fibrotic phase of the model. However, AF-1 (10 mg · kg^-1^ but not 2 mg · kg^-1^ or 5 mg · kg^-1^) (5 mg · kg^-1^ data not shown) alleviated the pulmonary fibrosis (Figure 4A - C). The AF-1 effective dose (10 mg · kg^-1^) for the therapeutic treatment was higher than that (2 mg · kg^-1^) in the preventive treatment. These results indicate that AF-1 has anti-inflammatory and antifibrotic actions in bleomycin-induced lung injury.

### The antiproliferative activity of AF-1 in TGF-β1-stimulated NIH3T3 cells

**Figure 5:**
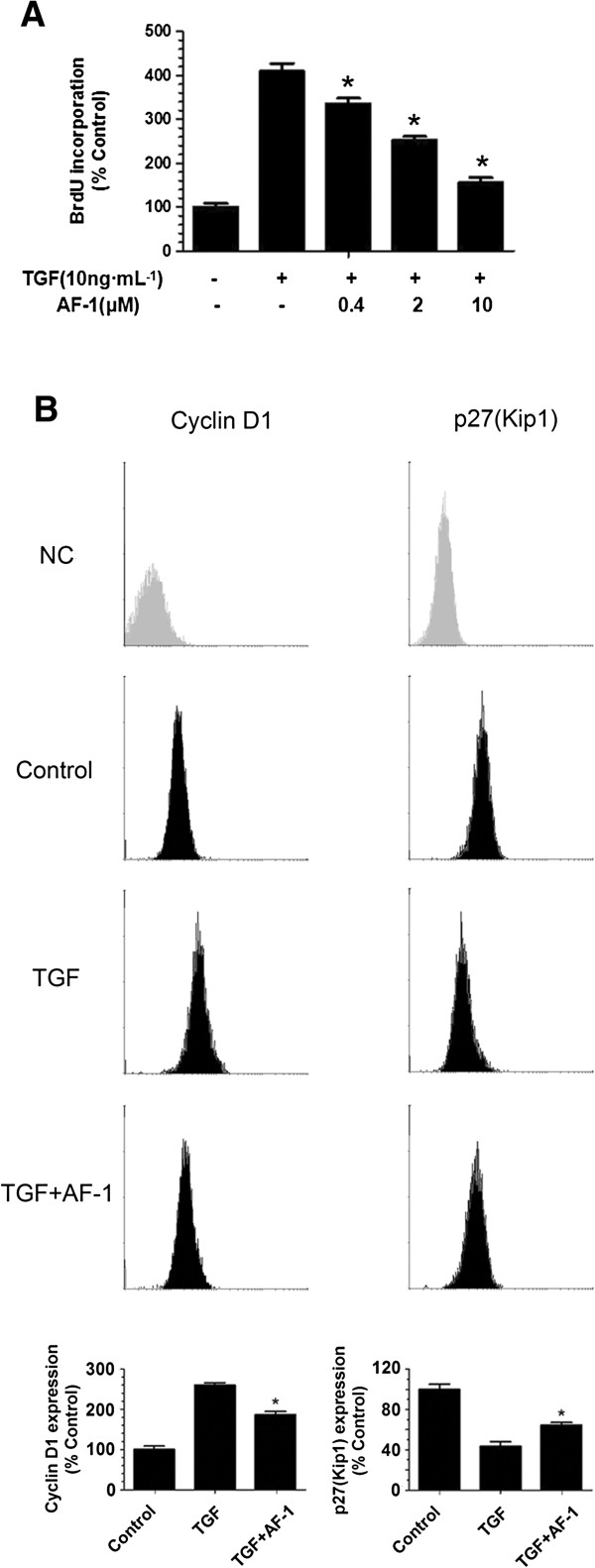
**AF-1 regulated the expression of cyclin and inhibited the growth of NIH3T3. (A)**. The cells were cultured in medium containing TGF-β1 (10 ng · mL^-1^) with various concentrations of AF-1 (0–10 μM). The OD values of BrdU incorporation were measured by microplate reader. **(B)**. The cells were cultured in medium containing TGF-β1 (10 ng · mL^-1^) in the absence or presence of AF-1 (10 μM), the mean fluorescence intensity (MFI) of Cyclin D1 and p27 were determined by flow cytometry. Before statistics and analysis, the data divided by the mean of control group. NC: negative control. n = 3–4. Bars: mean ± SD . *P ≤ 0.05 vs TGF group.

**Figure 6:**
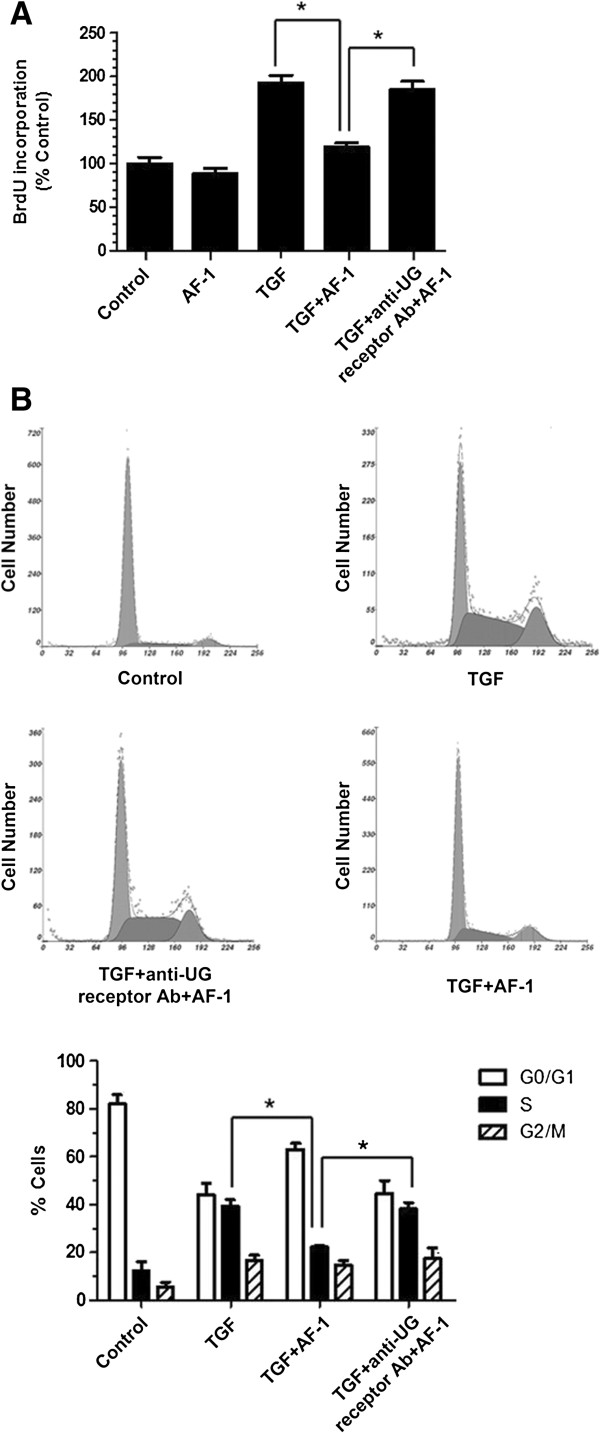
**AF-1 suppressed the NIH3T3 proliferation by interacting with UG receptor.** The cells were cultured in medium containing TGF-β1 (10 ng · mL^-1^) in the absence or presence of AF-1 (10 μM), and with or without anti-uteroglobin (UG) receptor antibody (50 μg · mL^-1^). Cell proliferation was assessed by BrdU incorporation **(A)** and flow cytometry **(B)**. Before statistics and analysis, the data divided by the mean of control group. n = 3. Bars: mean ± SD . *P ≤ 0.05 vs TGF group.

To test for antiproliferative activity of AF-1 *in vitro,* the incorporation of BrdU into NIH3T3 cells was measured. TGF-β1 (10 ng · mL^-1^) significantly induced BrdU incorporation into NIH3T3 cells, while AF-1 (0–10 μM) inhibited the effect of TGF-β1 in a concentration-dependent manner (Figure 5A). Cell cycle analysis also showed that the TGF-β1-induced increase in S phase entry was reversed by AF-1 (10 μM) (Figure 6B). We further quantified the expression of cyclin D1 and p27 by flow cytometry and found that TGF-β1 increased the expression of cyclin D1 and decreased the expression of p27. In contrast, after AF-1 (10 μM) treatment, the TGF-β1-induced changes to both cyclin D1 and p27 were reversed (Figure 5B).

### UG receptor plays an important role in the antiproliferative activity of AF-1

In our previous study, we showed that AF-1 specifically binds to the UG receptor and activates the mitogen-activated protein kinase (MAPK) signaling pathway [22]. Therefore, in the present study, we sought to determine whether the antiproliferative activity of AF-1 was related to the UG receptor. Both the BrdU incorporation assay (Figure 6A) and cell cycle analysis (Figure 6B) showed that the antiproliferative effect of AF-1 (10 μM) was reversed by pretreatment with anti-UG receptor antibody (50 μg · mL^-1^). This result suggests that the antiproliferative activity of AF-1 is mediated by the UG receptor.

## Discussion

The present study showed a role for AF-1 in the suppression of pulmonary fibrosis. This report is the first to demonstrate that AF-1 prevented bleomycin-induced inflammation and fibrosis in lungs. Furthermore, AF-1 significantly inhibited the TGF-β1-induced proliferation of NIH3T3 cells *in vitro* by interacting with the UG receptor.

The bleomycin animal model is the best available experimental tool for studying the pathophysiology of IPF and testing novel pharmaceutical compounds for IPF [23]. However, the IPF progression in humans differs the development of pulmonary fibrosis in the bleomycin-induced mouse model. In the mouse model, bleomycin first induces gross inflammation in the rodent lung, and this sustained damage leads to the initiation of fibrosis [24,25]. Numerous anti-inflammatory agents inhibit fibrosis in bleomycin animal models, but none of them has shown a comparable effect against IPF in humans [26]. Therefore, it is important to distinguish the anti-inflammatory and antifibrotic actions of test drugs in this model. If compounds only work when administered over the entire experimental period but are ineffective when administered after most of the inflammatory response is resolved, these compounds should only have anti-inflammatory activity. However, if drugs work irrespective of the treatment regimen, the drugs should have antifibrotic activity. In our study, we first performed a time course experiment to determine the duration of the inflammatory and fibrotic phases. After Day 10, the inflammatory cytokines significantly decreased, accompanied by a significant increase in collagen deposition (Figure 1). Therefore, we speculated that the course of pulmonary fibrosis in this bleomycin model changed from the inflammatory phase to the fibrotic phase after Day 10. Our results show a process of inflammatory and fibrotic phase development that is similar to previously published data, and our results further confirm that the bulk of the inflammatory response was resolved by approximately Day 10 after bleomycin injection [21]. Therefore, comparison of the preventive treatment, which began at the initiation of the inflammatory response (i.e., starting on Day 0), with the therapeutic treatment, which commenced after most of the inflammatory response was resolved (i.e., starting at Day 11), allowed us to distinguish the anti-inflammatory and antifibrotic actions of AF-1.

As a novel antiinflammatory peptide, AF-1 has highly potent anti-inflammatory activity in different animal models, tissues and organs [16-18]. In the present study, continuous treatment with AF-1 (2 mg · kg^-1^) starting on Day 0 significantly alleviated the inflammation on Day 7 and the collagen deposition on Day 28 in the bleomycin-induced model of lung injury (Figures 2 and 3). The anti-inflammatory activity of AF-1 in this model may be due to its blockade of neutrophil trafficking, suppression of the activation of macrophages and reduction of the production and release of inflammatory mediators [11,12].

Next, we examined the antifibrotic effects of AF-1 in the bleomycin mouse model. When therapeutic treatment with AF-1 began on Day 11, AF-1 (10 mg · kg^-1^ but not 2 mg · kg^-1^ or 5 mg · kg^-1^) (5 mg · kg^-1^ data not shown) significantly attenuated the collagen deposition on Day 28 (Figure 4). Our results clearly show that AF-1 alleviated pulmonary fibrosis significantly by both anti-inflammatory and antifibrotic actions, which were independent of the treatment regimen. However, the effective AF-1 dose for the therapeutic treatment was higher than that for the preventive treatment.

The proliferation and extracellular matrix (ECM) production of fibroblasts constitute the final common pathogenic factor in fibrotic diseases. Numerous antifibrotic strategies aim to target the activation, proliferation and/or recruitment of fibroblasts, which occur in response to the strengthened actions of profibrotic cytokines. TGF-β is one of the most important profibrotic cytokines and is produced by various types of cells, including macrophages, epithelial cells and fibroblasts. After being activated, TGF-β becomes a pleiotropic cytokine with proliferative and chemotactic properties [27]. As mentioned above, any drugs that successfully ablate the activation and/or proliferation of fibroblasts *in vitro* might have antifibrotic activity *in vivo*. Therefore, to further research the underlying mechanisms of AF-1, we observed the effect of AF-1 on TGF-β1-induced proliferation in NIH3T3 cells. We found that AF-1 inhibited the TGF-β1-induced proliferation in NIH3T3 cells in a concentration-dependent manner, and the inhibition induced by AF-1 might have been due to its regulation of the expression of cyclin D1 and p27 (Kip1) (Figure 5), which determine the transition from G0/G1 phase to S phase [28-30].

The mechanism responsible for the antiproliferative effect of AF-1 on TGF-β1-induced proliferation in NIH3T3 cells is not clear. Recently, we found that AF-1 specifically binds to a saturable membrane receptor, the UG receptor, and activates the MAPK signaling pathway [22]. Kundu and coworkers first identified the UG receptor in several types of cells; treating the cells expressing the UG receptor with UG dramatically suppressed cell growth and ECM invasion, but such treatment had no effect on cells lacking the UG receptor [5,31-33]. Subsequently, molecular cloning and analysis of the UG receptor cDNA indicated that it encodes a 55-kDa protein; on the basis of amino acid similarity, the UG receptor was classified in the lipocalin receptor family, which has remarkable functional diversity [34,35].

As a synthetic peptide derived from UG, AF-1 has similar activities to UG [10]. Because UG can inhibit cell growth and ECM invasion by interacting with the UG receptor, we sought to determine whether the antiproliferative activity of AF-1 was also related to the UG receptor. Our results show that the antiproliferative effect of AF-1 was reversed by pretreatment with anti-UG receptor antibody (Figure 6). Therefore, AF-1 might suppress TGF-β1-induced NIH3T3 proliferation by interacting with the UG receptor.

According to our studies, the antifibrotic effect of AF-1 in bleomycin-treated mice may be related to its antiproliferative activity, which is mediated by the UG receptor. Of course, the roles of the UG receptor in the development of pulmonary fibrosis *in vivo* require further rigorous investigation. Similarly to most drugs or the UG protein itself, AF-1 has an antifibrotic effect that is difficult to explain by a simple mechanism of action. Moreno reported that AF-1 can inhibit the activity of transglutaminase-2 (TG-2) [36]. TG-2 is a multifunctional protein that plays an important role in bleomycin-induced pulmonary fibrosis [37,38]. Overexpression of TG-2 increases fibronectin deposition and matrix organization *in vitro*. In contrast to wild-type mice, TG-2 knockout mice develop markedly less severe pulmonary fibrosis [37]. Therefore, inhibiting the activity of TG-2 by AF-1 may also be helpful in alleviating pulmonary fibrosis.

## Conclusions

In summary, our *in vitro* results indicate that AF-1 could inhibit fibroblast proliferation by interacting with the UG receptor, and our *in vivo* results indicate that AF-1 plays an anti-inflammatory and antifibrotic role in inhibiting bleomycin-induced lung injury. Thus, AF-1 has potential as a therapeutic tool for pulmonary fibrosis.

## Abbreviations

AF-1: Antiflammin-1; UG: Uteroglobin; BLM: Bleomycin; IPF: Idiopathic pulmonary fibrosis; HYP: Hydroxyproline; TGF-β1: Transforming growth factor-β1; TNF-α: Tumor necrosis factor-α; IL-1β: Interleukin-1β; MAPK: Mitogen-activated protein kinase; ECM: Extracellular matrix; BALF: Bronchoalveolar lavage fluid.

## Competing interests

The authors declare that they have no competing interests.

## Authors’ contributions

ZQL, SJY and DDF designed the study. WL, JW, YHH, YC, QMC and YL performed the experiments. WL, JW, JZH and CL performed the statistical analysis. WL wrote the manuscript. All authors read and approved the final manuscript.
